# A Mobile App Directory of Occupational Therapists Who Provide Home Modifications: Development and Preliminary Usability Evaluation

**DOI:** 10.2196/14465

**Published:** 2020-03-30

**Authors:** An Thi Nguyen, Emily Kling Somerville, Sandra Martina Espín-Tello, Marian Keglovits, Susan Lynn Stark

**Affiliations:** 1 Program in Occupational Therapy Washington University School of Medicine St. Louis, MO United States

**Keywords:** mHealth, mobile app, occupational therapist, occupational therapy, older adult, user-computer interface

## Abstract

**Background:**

Home modifications provided by occupational therapists (OTs) are effective in improving daily activity performance and reducing fall risk among community-dwelling older adults. However, the prevalence of home modification is low. One reason is the lack of a centralized database of OTs who provide home modifications.

**Objective:**

This study aimed to develop and test the usability of a mobile app directory of OTs who provide home modifications in the United States.

**Methods:**

In phase 1, a prototype was developed by identifying OTs who provide home modifications through keyword Web searches. Referral information was confirmed by phone or email. In phase 2, community-dwelling older adults aged older than 65 years and OTs currently working in the United States were purposefully recruited to participate in a single usability test of the mobile app, Home Modifications for Aging and Disability Directory of Referrals (Home Maddirs). Participants completed the System Usability Scale (SUS) and semistructured interview questions. Interview data were coded, and themes were derived using a grounded theory approach.

**Results:**

In phase 1, referral information for 101 OTs across 49 states was confirmed. In phase 2, 6 OTs (mean clinical experience 4.3 years, SD 1.6 years) and 6 older adults (mean age 72.8 years, SD 5.0 years) participated. The mean SUS score for OTs was 91.7 (SD 8.0; out of 100), indicating good usability. The mean SUS score for older adults was 71.7 (SD 27.1), indicating considerable variability in usability. In addition, the SUS scores indicated that the app is acceptable to OTs and may be acceptable to some older adults. For OTs, self-reported barriers to acceptability and usability included the need for more information on the scope of referral services. For older adults, barriers included high cognitive load, lack of operational skills, and the need to accommodate sensory changes. For both groups, facilitators of acceptability and usability included perceived usefulness, social support, and multiple options to access information.

**Conclusions:**

Home Maddirs demonstrates good preliminary acceptability and usability to OTs. Older adults’ perceptions regarding acceptability and usability varied considerably, partly based on prior experience using mobile apps. Results will be used to make improvements to this promising new tool for increasing older adults’ access to home modifications.

## Introduction

### Background

Difficulties performing activities of daily living (ADLs), such as bathing, dressing, or toileting, place older adults at increased risk for adverse outcomes, including poorer health and frailty, premature institutionalization, and mortality [1-4]. Approximately 30% of community-dwelling older adults have difficulty performing one or more ADLs [5,6]. With the number of Americans aged older than 65 years projected to rise from 49 million to 98 million between 2016 and 2060, the number of older adults living with ADL limitations is expected to surge [7]. Accordingly, *Healthy People 2020* outlined an urgent goal to reduce the adverse outcomes of daily activity limitations among older adults as a national health priority [8].

Evidence-based home modifications delivered by occupational therapists (OTs) are an effective intervention to improve older adult’s safety and independence when performing ADLs [9-16]. The goal of home modifications is to reduce environmental barriers in an older adult’s home to match declining physiological competencies associated with increasing age and medical conditions. Home modification interventions may include training older adults and caregivers to use compensatory strategies and adaptive equipment to facilitate safer performance and increased independence in ADLs [9,11]. Home modifications may also include recommendations for major structural changes to a home (eg, addition of grab bars or a curbless shower) and the removal of environmental hazards to reduce the risk of falls and prevent serious resulting injuries [17]. OTs are essential to evidence-based evaluation and delivery of home modifications because they possess the biomedical and psychosocial knowledge, skills, and training to accurately assess an older person’s physiological competencies (eg, cognitive, motor, and sensory functions), evaluate social and physical environmental barriers impeding ADL performance, identify home modifications that reduce the mismatch between personal competencies and environmental demands, train older adults and caregivers in the correct and safe use of home modifications, and assess intervention outcomes to ensure ADL limitations have been reduced [18]. In the United States, home modifications and the accompanying services provided by OTs are often privately funded, although grant funding may be available from state or local governments, public programs, or nonprofit organizations to help cover the cost of home modifications for low-income individuals [19].

However, many older adults continue to lack access to evidence-based home modifications, in part, because of the lack of information on OTs who provide home modifications [19-22]. Older adults, family members, caregivers, social service coordinators, and health care professionals may lack awareness of locally available OTs who can provide home modifications to help facilitate an older adult’s safe return home after hospital discharge or to promote aging in place [22]. This lack of information may delay and even preclude the delivery of home modifications when they are needed most to improve safety and independence in ADL performance and reduce the risk of long-term adverse health outcomes for older adults [12,19,22-24]. Directories of resources for home modifications exist, including the *National Directory of Home Modification and Repair Resources* and *Eldercare Locator* [25,26]. Existing directories, however, lack comprehensive referral information on OTs who deliver home modifications as part of their database of resources. Therefore, there is a need to develop a centralized, publicly accessible database of information on OTs who provide home modifications to increase intervention access, improve care coordination, and reduce care delivery delays for older adults who are discharged from health care facilities back to independent living and for those seeking to maintain independent living or age in place.

### Objectives

To address this challenge, this study sought to develop a mobile app as a centralized database containing referral information for OTs in the United States who provide home modifications and to preliminarily evaluate its acceptability and usability for OTs and older adults. In this paper, we present the methods and results of developing a prototype of the mobile app (phase 1) and usability testing to inform iterative improvements to the prototype (phase 2). The objective of the mobile app, named Home Modifications for Aging and Disability Directory of Referrals (Home Maddirs), is to aid older adults, family members, caregivers, social service coordinators, and health care providers in identifying local OTs who provide home modifications. We hypothesized that the mobile directory would be acceptable and usable to OTs and community-dwelling older adults.

## Methods

### Phase 1: Prototype Development

A previously published protocol for health-related directory development was adapted to develop a prototype of the mobile app [27]. To identify OTs for inclusion in the directory, keyword Web searches were conducted between October 2018 and March 2019 using Web search engines (Google and LinkedIn) for the following terms, where all 50 US states and Puerto Rico were included as search terms: (“home modification” OR “home assessment”) AND “occupational therapist” AND “[state/territory].”

For each search query, the first author (AN) reviewed the top 300 search results or the maximum number of search results returned, whichever was first reached, to identify OTs who provided home modifications for inclusion in the directory. Snowball sampling was used to identify additional OTs by soliciting referrals from (1) OTs previously identified through Web searches; (2) responses to posts on Web community forums belonging to the *American Occupational Therapy Association’s Home & Community Special Interest Section* and the *Home Modification Occupational Therapy Alliance* (HMOTA); and (3) cross-referencing two other existing resource databases related to home modifications, the *National Directory of Home Modification and Repair Resources* and *Eldercare Locator* [25,26].

Current clinical practice guidelines and a clinical reasoning guideline for the delivery of home modifications by OTs were used to define basic data fields and build search filters into the mobile app [28,29]. Basic data fields for referral sources included organization or business name, address, telephone number, email, website, specific populations served (eg, children and older adults), home modification services provided (eg, home evaluation, consultation, construction, project management, and caregiver training), payment methods or insurances accepted, and languages available for service provision. The names of businesses or organizations were incorporated, instead of the names of individual providers, to improve sustainability by reducing the impact of provider turnover. A built-in form to collect submissions from app users for new database entries and updates to current entries was also added to facilitate future updates to the directory. Information for each data field was initially retrieved from publicly available information online. Referral information was confirmed by self-report over email or phone call with each therapist.

### Phase 2: Usability Testing

#### Participants

To evaluate the preliminary acceptability and usability of the mobile app, usability tests were conducted with community-dwelling older adults and OTs as targeted end user groups. Older adults (n=6) and OTs (n=6) were recruited by purposeful sampling from a list of local contacts obtained from clinical research coordinators at the Participation, Environment and Performance Laboratory and the Community Practice Clinic at the Washington University School of Medicine (St Louis, Missouri, USA). A sample size of 8 to 10 is recommended to detect 80% of usability problems [30].

#### Inclusion Criteria

Community-dwelling older adults were recruited if they (1) were aged 65 years or older, (2) could speak English, (3) could live independently in a noninstitutionalized setting, and (4) self-reported no health concerns about using a mobile app other than lack of experience. OTs were included if they (1) could speak English and (2) currently worked as a licensed OT in the United States (part time, full time, per diem, or self-employed).

#### Exclusion Criteria

OTs not currently working were excluded to retrieve feedback regarding app acceptability and usability informed by current clinical practice experience.

#### Usability Testing Procedures

The authors asked the institutional review board (IRB) at the Washington University School of Medicine in St Louis to review all study procedures, and the IRB verified that the study qualified for IRB exemption as a quality improvement initiative (IRB study ID number: 201901022). Participants were screened by phone or email to assess eligibility and coordinate attendance at a single usability testing session. Older adults and OTs participated individually in a single, 45-min test session. All usability tests were performed in a naturalistic setting [31]. Older adults were visited in their home, whereas OTs were visited in their clinical workplace setting to conduct all testing procedures. This approach eliminated the need for older adults to access transportation (supporting inclusive recruitment of older adults with a wider range of physical capabilities and socioeconomic backgrounds) and accommodated clinicians’ busy work schedules.

Verbal consent was obtained from all participants at the start of each session after explaining its purpose and structure. A script was read aloud to describe the general purpose of the mobile app, but no further instructions were provided on how to use the app. Participants were instructed to perform a set of five task scenarios using the app on a mobile tablet device (Apple iPad 4). Tasks scenarios consisted of representative tasks expected to be typically performed by end users. These tasks were to (1) identify the name of the OT nearest to your current location who provides home modifications, (2) identify the name of the home modification funding source nearest to your current location, (3) search for the list of all OTs within 200 miles of your current location who provide home modifications, (4) search for the list of all home modification funding sources within your state, and (5) search for the list of all home modification funding sources for people with low income in your state. In addition to identifying OTs, referral information for funding sources to receive financial assistance for home modifications was also incorporated into the directory and was tested in tandem during usability testing. Instructions for each task were provided orally and in writing.

A concurrent think-aloud protocol was used to obtain insights into usability problems that participants experienced [30,32-34]. Participants were instructed to simultaneously verbalize their mental thought processes as they performed each task scenario. The test administrator (AN) was not allowed to provide assistance during tasks and was only allowed to use one of the two probes during the test session: (1) “Keep talking” after 15 seconds of silence to encourage participants to continue verbalizing their thoughts and (2) “Um-hum,” “oh,” or “okay,” to affirm active listening [34]. The maximum amount of time allowed for each task scenario was 5 min, after which the participant was instructed to move on to the next task.

#### Outcome Measures

A mixed method approach was used to assess primary outcomes of acceptability and usability of Home Maddirs with the following outcome measures: (1) *task accuracy* (the rate of successful task completion calculated as the number of tasks completed successfully divided by the total number of tasks undertaken × 100), (2) *task efficiency* (time to complete each task in seconds, starting from the time the participant finishes receiving instructions to the time they found their answer and finished reviewing it), (3) *error rate* (number of errors per task, where errors are defined as unintended actions, such as miss clicks), (4) *types of errors* (qualitative descriptions of errors), and (5) *perceived task difficulty* (immediately after each task scenario, participants were asked, “Overall, how difficult or easy did you find this task?”; they responded using a 7-point Likert scale, ranging from 1=very difficult to 7=very easy) [35].

#### System Usability Scale

The System Usability Scale (SUS) is a valid and reliable 10-item questionnaire that has been used extensively to evaluate the usability of a wide range of technologies, systems, and services, including mobile apps [36]. The SUS was further selected as a usability measure because of the ease with which participants would be able to understand its questions in the context of the study’s usability testing scenarios. Questions on the SUS are rated on a 5-point Likert scale, ranging from 1=strongly disagree to 5=strongly agree, and summed to generate a total usability score. Total usability scores on the SUS range from 0 to 100, where higher scores indicate greater acceptability and usability (ie, greater ease of use, ease of learning to use, and self-confidence in using the mobile app). SUS scores below 70 indicate a mobile app considered to be unacceptable by respondents, whereas scores above 70 indicate good acceptability and usability, and scores above 90 indicate excellent usability [36,37].

#### Qualitative Interview Data

Older adults’ and OTs’ subjective evaluations of acceptability and usability of the mobile app were collected through responses to open-ended interview questions. A semistructured interview guide was developed by the research team to obtain qualitative feedback on barriers and facilitators to acceptability and usability. Interview questions included (1) “What made it easy or difficult for you to use the app?,” (2) “What did you like or dislike about the app design?,” and (3) “What could be changed to make it easier for you to use the app?.” OTs were additionally asked questions to probe for barriers and facilitators to adoption of the mobile app in their clinical practice setting. These questions included (1) “Could you foresee yourself or others using the app in your practice setting?,” (2) “What difficulties do you foresee with using the app in your practice setting?,” and (3) “What would make it easier for professionals to use the app in your practice setting?.”

#### Demographics

Older adults self-reported their age, gender, marital status, race/ethnicity, and education. OTs self-reported their age, gender, race/ethnicity, education, current clinical practice setting, and years of clinical work experience. Both older adults and OTs were asked to rate their prior extent of mobile app usage measured using three items adapted from the smartphone usage subscale of the Media and Technology Usage and Attitudes Scale and on a 6-point scale with the following question: “In the last month, how much time did you spend using mobile apps?,” which was rated from 1=less than 1 hour, 2=1 to 2 hours, 3=2 to 4 hours, 4=4 to 6 hours, 5=6 to 10 hours, to 6=more than 10 hours [38,39].

#### Data Analysis

All usability test sessions were audiotaped, and mobile device screens were screen recorded throughout testing. All data were deidentified before storage and analysis. Descriptive statistics of participant demographics and quantitative measures of acceptability and usability were calculated using SPSS version 24.0 (IBM, New York, USA). Qualitative interview responses were transcribed verbatim. The first author (AN) coded all qualitative interview data using content coding analysis. A constant comparative method based on the grounded theory approach was used so that interview transcripts were continually reevaluated for themes emerging from consistencies and differences in coded terms [40-44]. Themes were clustered into categories of barriers and facilitators to acceptability and usability. Categories, themes, and their associated codes were developed and documented using NVivo version 12.0 (QSR International, Melbourne, Australia). Member checking was used to enhance the trustworthiness of findings, whereby themes were shared with participants by phone or email for respondent validation [45,46].

## Results

### Phase 1: Prototype Development

In total, 148 prospective directory entries were identified from keyword Web searches, responses to online community forum posts, and snowball sampling. Of these, 118 prospective entries responded to outreach by email or phone (80% response rate). Referral information for 101 OTs was confirmed and incorporated into the mobile directory. Reasons for which prospective entries responded but were not included in the directory were as follows: seven organizations that employ OTs who do not provide home modifications, five organizations that do not employ OTs (eg, they were solely home builders), 4 OTs who had retired from providing home modifications, and 1 OT who had not yet started providing home modifications but was planning to do so in the near future.

**Figure 1:**
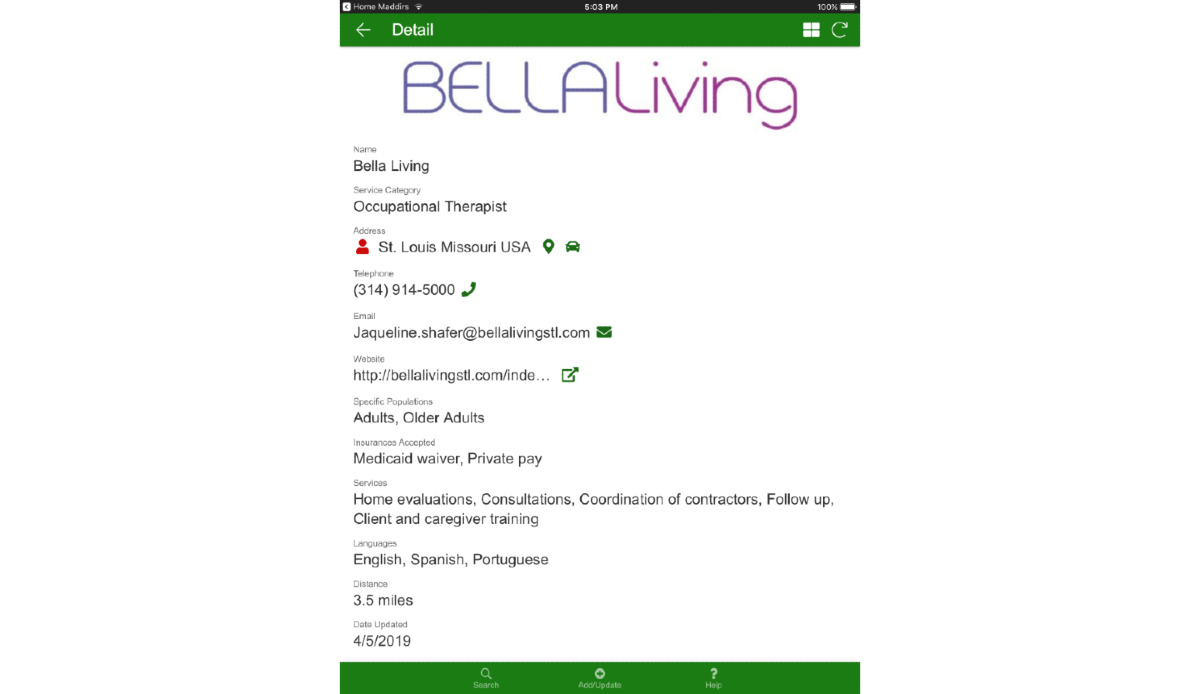
Home Modifications for Aging and Disability Directory of Referrals user interface displaying a single directory entry.

The app uses geolocation services on a mobile device to curate referral information based on geographic distance from the app user and other relevant decision-making factors selected, such as insurance or payment methods accepted, patient populations served, and the scope of home modification services provided. Figure 1 shows an example of how an OT’s provider information is displayed as an entry in the directory, which includes their business name, business address, business telephone number, business email, business website, specific patient populations served (eg, children, adults, or older adults), insurances accepted, home modification services provided (eg, consultation, home evaluation, coordination of contractors, and follow-up on contractors’ work), languages in which services are provided, and distance from the location of the mobile device to the provider’s address.

### Phase 2: Usability Testing

#### Demographics

Demographics of OTs and older adults who participated in usability testing are shown in Table 1. OTs reported currently working in a wide range of clinical practice settings, including acute care (n=1), inpatient rehabilitation (n=2), outpatient/community practice clinic (n=2), and private practice specifically providing home modifications (n=1). Therapists’ mean clinical experience was 4.3 (SD 1.6) years. Participants’ prior extent of mobile app usage is shown in Table 2.

**Table 1:** Demographics of participants who participated in usability testing.

Characteristic			Occupational therapists (n=6)		Older adults (n=6)
**Age (years)**					
	Mean (SD)	35.7 (9.8)		72.8 (5.0)	
	Range	28-54		67-81	
**Gender, n (%)**					
	Female	6 (100)		6 (100)	
**Race, n (%)**					
	White	6 (100)		1 (17)	
	African American	0 (0)		5 (83)	
**Education, n (%)**					
	High school/general educational development	0 (0)		1 (17)	
	Some college	0 (0)		2 (33)	
	College degree	6 (100)		3 (50)	
**Marital status, n (%)**					
	Married	N/A^a^		1 (17)	
	Single	N/A		3 (50)	
	Widowed	N/A		2 (33)	

^a^N/A: not applicable.

**Table 2:** Participants’ extent of prior mobile app usage.

Media and Technology Usage and Attitudes Scale question^a^			Occupational therapists (n=6)		Older adults (n=6)
**How often do you search for information on a mobile phone/tablet, n**					
	Never	0		3	
	Several times a week	0		2	
	Several times a day	4		0	
	Once an hour	1		0	
	All the time	1		1	
**How often do you get directions or use GPS on a mobile phone/tablet, n**					
	Never	0		2	
	Once a month	0		3	
	Several times a month	0		1	
	Once a week	1		0	
	Several times a week	3		0	
	All the time	2		0	
**How often do you use apps for any purpose on a mobile phone/tablet, n**					
	Never	0		2	
	Once a week	0		1	
	Several times a week	0		1	
	Once a day	0		1	
	Several times a day	3		0	
	Several times an hour	1		0	
	All the time	2		1	
**In the last month, how much time did you spend using apps for any purpose on a mobile phone/tablet, n**					
	<1 hour	1		3	
	2-4 hours	0		1	
	4-6 hours	0		2	
	>10 hours	5		0	

^a^All response categories for each question are not listed; only those that received at least one participant response are listed.

#### Quantitative Outcomes

Quantitative usability metrics are shown in Table 3. Percent task completion ranged from 0% to 100% for older adults. Specifically, 2 older adults were unable to successfully complete any task scenarios—one consistently took longer than the allowed 5 min per task, whereas the other attempted but gave up early on tasks citing that it was too difficult. Compared with other older adult participants, these 2 older adults were observed to be older and had less prior experience using mobile apps. Their data were excluded from the calculation of older adult’s average task efficiency but included in all other usability measures.

**Table 3:** Quantitative usability metrics.

Measure	Occupational therapists (n=6), mean (SD)	Older adults (n=6), mean (SD)
System Usability Scale score	91.7 (8.0)	71.7 (27.1)
Percent task completion	93 (16)	60 (49)
Average task efficiency^a^ (seconds per task)	40.7 (32.2)	97.5 (57.4)
Average error rate (errors per task)	0.7 (0.5)	1.9 (1.9)
Average task perceived difficulty (1=very difficult and 7=very easy)	6.6 (0.4)	4.6 (2.2)

^a^Excludes instances of task scenarios that were not completed successfully.

#### Qualitative Outcomes

Qualitative themes derived from screen recordings and interview transcripts are summarized in Table 4.

#### Error Types

Errors experienced by both OTs and older adults included miss clicks on various app features and the addition of search filters that overly limited search results (eg, selecting to filter by both the state and the geographical distance from the user when asked to search for all resources in one’s state). Older adults additionally experienced more frequent and diverse types of errors, including difficulty understanding how to initiate the search function, difficulty accurately interpreting the meaning of search results (eg, not knowing which search result was geographically closest to them despite distances being labeled), difficulty scrolling on a touch screen device, and difficulty remaining oriented while scrolling or navigating between views within the app.

**Table 4:** Qualitative themes of errors, barriers, and facilitators of acceptability and usability.

Category	Occupational therapists	Older adults
Error types	Miss clicks within app Extra search filters added to search query	Miss clicks within app Extra search filters added to search query Difficulty initiating search function Difficulty interpreting search results Difficulty scrolling using touch screen Difficulty navigating between views within the app
Barriers to acceptability and usability	Need for more information on scope of referral services	High cognitive load of user interface Need to reduce jargon Lack of operational skills Need to accommodate age-related sensory changes
Facilitators of acceptability and usability	Perceived usefulness Social support (ie, technical support guidance) Time to practice to gain familiarity Multiple options to access information	Perceived usefulness Social support (ie, assistance from family, caregivers, and health care providers) Time to practice to gain familiarity Multiple options to access information

#### Barriers to Acceptability and Usability

Barriers to acceptability and usability conveyed by OTs included the need for more information regarding the scope of home modification services provided by referrals listed in the directory:

I guess I can go in and click online but if it had just a little list of some of the things that they do from their information page so I can quickly decide if it fits.

Occupational therapist 2

Barriers experienced by older adults included high cognitive load presented by the prototype’s user interface. For example, older adults commented that the presence of a map accompanying search results added unnecessary complexity:

I don’t mind the map being there but I don’t see the reason for the map being there...I think the map is fine, I just, it’s kind of distracting because I’m looking for something on the map when I could have just gone over here [to the other side of the screen].

Older adult 1

To reduce cognitive load, older adults also pointed to the need to reduce jargon and use terminology that resonates with consumer needs and services they would seek:

Definitely the services, you know, you have to phrase them in such a way that it’s something like – “Oh yeah, I think that’s something I need.”

Older adult 5

The majority of older adults pointed to their lack of experience using mobile devices and apps as a barrier. They emphasized their limited skills to operate mobile apps in general, difficulty defining an efficient search strategy to search the directory, and needing more instruction on how to use the app initially:

If I had [a mobile device] that I could just get all to myself and maybe have some kind of booklet that I could read to learn, you know. Some instruction, you know.

Older adult 4

Older adults also suggested the need for design changes to increasingly accommodate age-related sensory changes, for example, increasing font size to accommodate decreased near visual acuity:

The writing was too little. I usually have to increase the size of the screen. This is too little for me.

Older adult 3

#### Facilitators of Acceptability and Usability

Most OTs and older adults perceived the mobile app to be useful as a facilitator of acceptability and usability:

Yeah, like right now, I feel like some of those referrals, we never were making them. Like we can tell patients to follow up on them or we’ll tell case management and they’ll try to get some of that set up for the discharge process, but I think because of the lack of knowledge of where to send them, sometimes those people might unintentionally fall through the cracks, so this would be a really nice tool for them to easily access where they could send them or increase their accessibility to that information to potentially help for discharge.

Occupational therapist 5

The information that I was looking for was clear in order to get to where I wanted to be, you know. So, I found the occupational therapist and that’s what I needed, and I found it, you know. And the contact was right there, all I had to do was call.

Older adult 2

Both OTs and older adults cited social support and time to practice to gain familiarity with the app as effective means to enhance acceptability and usability. For example, OTs stated that social support in the form of additional technical support would be useful:

I think the “Help” section should be like “I’m stuck what do I do?” Tech stuff like “I’m stuck, I can’t find what I’m looking for,” or maybe adding in there like what if my internet connection isn’t working. I mean you assume that they would have some idea of how to use the tablet, but those are the things I would include for the user maybe things less about OT and more about how to use the app or troubleshooting like to turn on your “location services” on your tablet or phone so that way you have a way to fix the problem if there’s some technical problem.

Occupational therapist 2

In contrast, older adults stated that social support from another person (eg, family member, caregiver, or health care professional) would be beneficial:

I would have to have a teacher, you know...then each day, I could practice what I learned from my instruction.

Older adult 4

Finally, both OTs and older adults suggested that multiple options to access information (eg, on a mobile device or a computer) would facilitate acceptability and usability. OTs specifically stated that having options to access information on either a mobile device or a computer would increase use by offering flexibility to accommodate diverse clinical environments and workflows:

We do have iPads at our disposal but I don’t know if it’s something that can also be done – I more often have my laptop than my iPad so I don’t know if it’s something that could be accessible through both.

Occupational therapist 5

In contrast, older adults stated that accessing the directory on a website with a computer mouse and keyboard would help facilitate usage because of the greater ease of navigation using a mouse compared with the touch screen:

You need a mouse real bad...the touch screen thing I just don’t like it.

Older adult 3

## Discussion

### Principal Findings

The principal findings of this study are the development of a centralized database of OTs who provide home modifications that is accessible as a mobile app, Home Maddirs, and preliminary evidence to suggest that the mobile app is acceptable and usable to OTs. OTs who participated in the study worked in a wide range of clinical practice settings and generally perceived the app to be easy to use and useful for increasing access to referral information. Mobile health (mHealth) apps are increasingly being used by health care providers to improve clinical workflow efficiency and as novel interventions to improve diverse health outcomes for patients [47-49]. Previous studies have explored the acceptability and usability of mHealth apps for OTs to facilitate clinical decision making for assistive equipment provision, movement activities for children with disabilities, and wheelchair training [50-52]. To the authors’ knowledge, this is the first study to develop and preliminarily evaluate an mHealth solution for increasing access to occupational therapy services related to home modifications.

Usability testing further suggested that the mobile app may be acceptable and usable to some older adults but that considerable variation exists among older adults’ perceptions. Although some older adults found the mobile app easy to use, others perceived it to be difficult to use, which appeared to be influenced by the older adult’s prior experience and comfort with using mobile apps. Previous studies have shown promising results demonstrating the acceptability of mHealth interventions among community-dwelling older adults [48,53]. Acceptability and usability of mHealth interventions, however, are attenuated by older adults’ prior experience using mobile apps, which influences their self-efficacy toward using mobile apps [54,55]. Qualitative themes arising from our interviews of older adults are consistent with the literature that suggests older adults, particularly those with less experience using mobile apps, would benefit from social support from caregivers and health care professionals to promote adoption and engagement with mHealth interventions [54,56,57]. Qualitative themes from this study further suggest that having multiple options to access referral information, such as on a desktop computer’s internet browser, in addition to the current mobile app platform, would increase the utilization of referral information by both older adults and clinicians based on individual preferences for using either interface during daily living or work routines. The choice to develop a progressive Web app that is delivered through the Web (which Home Maddirs is) may thus be an attractive option for other mHealth interventions to flexibly allow for dissemination simultaneously on mobile devices and internet browsers accessed on desktop computers.

When performing representative tasks using the mobile app, older adults, on average, perceived tasks to be more difficult, made more errors, encountered more diverse types of errors, and were less accurate and efficient compared with OTs. These observations point to the need for design improvements to better accommodate age-related changes; these include cognitive changes, such as decreased information processing speed and working memory capacity among older adults, and sensory changes, such as decreased near visual acuity and efficiency of visual information processing [58-61]. These results may also be partially explained by the lack of a requirement for participants to self-report comfort or competency with using mobile apps as part of the inclusion criteria for usability testing. The authors chose to embrace an ecological perspective by including older adults with less experience using mobile apps to increase the likelihood of uncovering usability problems that would be encountered in the general population of older adults, who, on average, have less experience using mobile apps compared with younger age groups [62].

### Limitations

The limitations of the study included a small sample size and lack of objective health screening that resulted in the recruitment of a nonrepresentative sample of OTs and community-dwelling older adults. Females and individuals with a minimum of a high school education were overrepresented in the sample. Furthermore, older adult participants self-reported no health concerns that would impair their ability to use mobile apps, but this self-report may have been inaccurate, and the results will not generalize to older adults with more significant health or functional impairments. Consequently, results can be used to provide insights to make iterative improvements to the app but should be interpreted with caution, as they are not likely to be reliable and generalizable to the entire population under study.

Other limitations included the use of a single coder to qualitatively code transcripts of audio recordings of usability test sessions. A minimum of two coders is suggested to improve veracity and trustworthiness of themes yielded from content coding analyses [41,43]. The decision to use a single coder was chosen because a primarily objective of this preliminary usability evaluation was to obtain insights to make improvements to the app. Future studies should use two coders to improve the strength of confidence in qualitative findings.

All usability testing sessions were conducted in a naturalistic setting, instead of a standardized laboratory environment. This may have reduced internal validity through the influence of differences in uncontrolled environmental variables within each unique testing environment. We attempted to standardize parts of the testing environment by having participants test the app on the same mobile tablet device running on the same wireless internet hotspot. The choice to conduct usability test sessions in a naturalistic environment was chosen because of the nature of target users groups being busy working clinicians or older adults, the latter of whom may lack reliable transportation or the physical capacity for travel.

### Conclusions

Home Maddirs is a promising new mobile directory tool to help increase older adults’ access to OTs who provide home modifications. This study provides preliminary evidence that the mobile app is acceptable and usable to OTs. The results of this study will be used to make improvements to the app, including design changes to accommodate age-related cognitive and sensory changes and to increasingly tailor views of information by audience (ie, consumer vs health care professional). Older adults’ perceptions of acceptability and usability of the mobile app varied considerably. To improve older adults’ access to the mobile directory information, caregivers, health care professionals, and social service coordinators should seek to provide social support, and multiple ways to access information should be made available for older adults who lack experience using mobile apps.

A working prototype of the mobile app is freely available online for public use [63]. Future work will seek to better understand the acceptability and usability of Home Maddirs for key stakeholder groups, including a broader, more representative sample of older adults, caregivers, social service coordinators, and interdisciplinary members of older adults’ health care teams. Future studies are further needed to explore the clinical utility of the mobile app, including comparisons between the use of the mobile app with other methods of accessing this information, optimum integration of its usage into clinical workflows, and evaluation of its impact on timely access to home modifications for older adults.

## Abbreviations

ADL: activity of daily living
HMOTA: Home Modification Occupational Therapy Alliance
Home Maddirs: Home Modifications for Aging and Disability Directory of Referrals
IRB: institutional review board
mHealth: mobile health
OT: occupational therapist
SUS: System Usability Scale
